# LONGITUDINAL ASSOCIATION OF DIGITAL HEALTH TECHNOLOGY USE WITH DEMENTIA IN OLDER ADULTS

**DOI:** 10.1093/geroni/igae098.1081

**Published:** 2024-12-31

**Authors:** Wenting Peng, Yuqian Luo, Minhui Liu

**Affiliations:** University of Washington, Seattle, Washington, United States; Hong Kong Polytechnic University, Hong Kong, Hong Kong; Ningxia Medical University, Yinchuan, Ningxia, China

## Abstract

Dementia is a global public concern and is associated with many adverse outcomes, such as disability. However, it remains unknown whether common modalities of digital health technology are associated with dementia. Using the data from 2015 to 2019 of the National Health and Aging Trends Study (NHATS), the study examined whether prior-year digital health technology use predicted dementia in a later year in community-dwelling older adults aged 65 years and older (N=4,002). Digital health technology use was ascertained with four items: filling prescriptions, contacting clinicians, handling insurance, and obtaining health information. Dementia was assessed using a modified NHATS definition, including a physician diagnosis, cognitive performance in three domains, and the AD8 Dementia Screening Interview. Lagged generalized estimation equation models were used to examine the longitudinal associations, adjusting for demographics and health-related factors. Seniors using any digital health technology in the prior year were at a lower risk of having possible or probable dementia in the later year (OR=0.61, 95% CI=0.51-0.74) than those not. Filling prescriptions, contacting clinicians, and obtaining health information statistically significantly predicted possible or probable dementia, with ORs of 0.61, 0.69, and 0.66, respectively. Additionally, any digital health technology use (OR=0.65, 95% CI=0.50-0.82), filling prescriptions (OR=0.60, 95% CI=0.43-0.85), and obtaining health information (OR=0.74, 95% CI=0.56-0.96) decreased the risks of having probable dementia. These findings suggest that digital health technology use is a protective factor of dementia. Intervention programs to delay dementia progression should incorporate strategies to help older adults use digital health technology in their daily lives.

